# Aniline Treatment of Phthisis

**Published:** 1887-09

**Authors:** 


Aniline Treatment of Phthisis.—Kremiansky's treatment was submitted by the Moscow Congress to investigation. The committee, consisting of Drs. Ostrumuff, Subertin, Shervinski, Klein, Voght and Bogoslovski, after a number of experiments subcutaneously and by inhalation, condemned the treatment and refused to make further investigation of the drug. Symptoms of paralysis of the respiratory center occurred even when given in very small quantities. It caused extreme disgust and appeared to be utterly useless from a therapeutic point of view.—*Technics.*

